# Paroxysmal Nocturnal Hemoglobinuria in Pregnancy Treated With Pegcetacoplan: Case Report and Pharmacokinetic Analysis

**DOI:** 10.1002/jha2.70179

**Published:** 2025-11-19

**Authors:** Benjamin Chin‐Yee, Benjamin D. Hedley, Rui Su, Barbra de Vrijer, Facundo Garcia‐Bournissen, Sarah Parkinson, Cyrus C. Hsia, Matthew Nichols, Ian Chin‐Yee, Christopher J. Patriquin

**Affiliations:** ^1^ Department of Pathology and Laboratory Medicine Western University London Ontario Canada; ^2^ Division of Hematology, Department of Medicine Western University London Ontario Canada; ^3^ Department of Pathology and Laboratory Medicine London Health Sciences Centre London Ontario Canada; ^4^ Department of History and Philosophy of Science University of Cambridge Cambridge UK; ^5^ Department of Obstetrics and Gynecology Western University London Ontario Canada; ^6^ Division of Pediatric Clinical Pharmacology Department of Pediatrics Western University London Ontario Canada; ^7^ Division of Hematology University Health Network, University of Toronto Toronto Ontario Canada

**Keywords:** case report, paroxysmal nocturnal hemoglobinuria, pegcetacoplan, pharmacokinetic, pregnancy

## Abstract

There is an unmet clinical need for effective treatment of paroxysmal nocturnal hemoglobinuria (PNH) in pregnancy for patients with inadequate response to C5 inhibitors. We report the first case of pegcetacoplan use in pregnancy with accompanying pharmacokinetic analysis. In a patient with transfusion‐dependent anemia on eculizumab, third‐trimester initiation of pegcetacoplan led to hematological stabilization, transfusion independence, and an uncomplicated term cesarean delivery of a healthy infant. Pegcetacoplan was undetectable in cord blood and breast milk despite therapeutic maternal levels, suggesting fetal and neonatal safety due to lack of significant placental and lactational transfer, and advancing evidence for pegcetacoplan use in pregnancy.

**Trial Registration**: The authors have confirmed clinical trial registration is not needed for this submission.

## 1 Introduction

Paroxysmal nocturnal hemoglobinuria (PNH) is a rare, acquired clonal hematological disorder caused by somatic *PIGA* mutations, resulting in deficiency of glycosylphosphatidylinositol‐anchored complement regulatory proteins and complement‐mediated intravascular hemolysis. PNH affects individuals of all ages [1] and is associated with life‐threatening complications if untreated [2], especially in pregnancy, where maternal mortality has historically approached 20% [3]. The C5 inhibitor eculizumab transformed PNH treatment by controlling intravascular hemolysis, reducing thrombotic risk, and improving survival and quality of life [4, 5]. Eculizumab substantially reduces maternal and fetal complications in pregnancy [6], and has become the standard of care for pregnant patients with PNH (where available and when indicated).

Despite established efficacy, up to one‐third of patients on C5 inhibitors remain transfusion‐dependent, often due to C3‐mediated extravascular hemolysis from persistent upstream complement activation [7, 8]. Proximal complement inhibitors offer treatment options for such patients, improving hematological and clinical outcomes in recent trials [9, 10, 11].

Pegcetacoplan, a first‐in‐class C3/C3b inhibitor, has shown superior efficacy in improving clinical and hematological outcomes in patients with persistent anemia on eculizumab [9] and in complement inhibitor‐naïve patients [12]. However, evidence for use in pregnancy remains limited [13]. We report the first case of pegcetacoplan use in pregnancy with accompanying pharmacokinetic data on placental and breast milk transfer, offering insights into the safety and feasibility of pegcetacoplan for the treatment of PNH in pregnancy.

## 2 Methods

### 2.1 Patient

The patient was a 24‐year‐old woman diagnosed with hemolytic PNH 5 years before her first pregnancy, with a 95% PNH neutrophil clone by flow cytometry. Upon diagnosis, she was initiated and maintained on eculizumab but remained transfusion‐dependent (∼1 unit RBC/month) despite dose escalation to 1200 mg intravenous every 11 days. Bone marrow showed normal trilineage hematopoiesis without evidence of dysplasia or aplastic anemia. She enrolled in a clinical trial of vemircopan (Factor D inhibitor), which was terminated early by the sponsor, and then received iptacopan (Factor B inhibitor), which was discontinued due to persistent nausea unresponsive to antiemetics. She subsequently transitioned to pegcetacoplan 1080 mg subcutaneous twice‐weekly and achieved transfusion independence with hemoglobin stabilization at approximately 110 g/L.

An unplanned pregnancy was identified during treatment with pegcetacoplan. Given the lack of safety data, she was reloaded with eculizumab at 4 weeks’ gestation, with pegcetacoplan discontinued at the time of the first eculizumab infusion. Prophylactic‐dose dalteparin was prescribed. Despite increased eculizumab dosing (1200 mg every 11 days), she redeveloped transfusion‐dependent anemia with signs of extravascular hemolysis, requiring 2–3 units RBC/month. Due to increasing transfusion requirements throughout the second trimester, following multidisciplinary consultation and shared decision‐making, twice‐weekly pegcetacoplan was reinitiated at 28 weeks’ gestation with a 4‐week overlap with eculizumab as per standard protocol [9]. Informed consent was obtained for off‐label use in pregnancy, pharmacokinetic sampling, and case reporting. Institutional ethics board review exemption was granted for this single‐patient case report.

### 2.2 Clinical Outcomes

Maternal and fetal outcomes were assessed throughout the third trimester and postpartum. Hematological response was evaluated using serial measurements of hemoglobin, hemolytic markers, and transfusion requirements. Complement levels/activity were measured using immunoturbidimetric assays for C3/C4 (Roche Diagnostics, Germany), and 50% hemolytic complement (CH50) by turbidimetric assay (Optilite, Binding Site, UK). Maternal clinical monitoring included evaluation for breakthrough hemolysis, thrombotic events, and other adverse events. Neonatal outcomes included gestational age at delivery, mode of delivery, birth weight, Apgar scores, and any complications identified during birth, hospitalization, or early follow‐up.

### 2.3 Pharmacokinetic Analysis

Maternal blood samples were obtained during routine collections pre‐ and postpartum. Cord blood samples were obtained from the umbilical vein at the time of delivery. Breast milk samples were collected on postpartum Days 1 and 2. Quantitative analysis of pegcetacoplan concentrations in maternal plasma, umbilical cord blood plasma, and breast milk was performed by an independent, accredited laboratory (Q^2^ Solutions BioSciences, USA) using a validated liquid chromatography‐tandem mass spectrometry method incorporating pegcetacoplan‐d_22_ as internal standard, with lower limits of quantification of 10 and 20 µg/mL for plasma and breast milk, respectively.

## 3 Results

### 3.1 Clinical Outcomes

**FIGURE 1:**
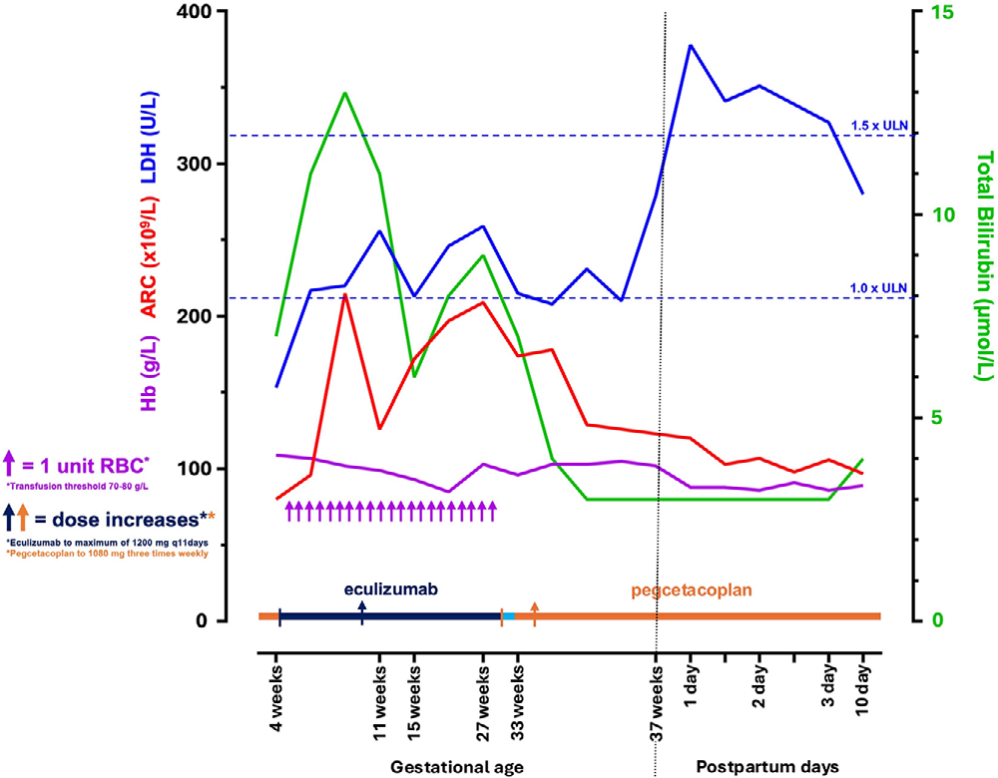
Hematological response to pegcetacoplan during pregnancy and postpartum. Serial measurements of hemoglobin (Hb, g/L, purple), absolute reticulocyte count (ARC, × 10^9^/L, red), lactate dehydrogenase (LDH, U/L, blue), and total bilirubin (Bili, µmol/L, green) are shown from early pregnancy through 10 days postpartum. Vertical arrows denote dose increases (blue for eculizumab, orange for pegcetacoplan) and red blood cell transfusions (purple), with a transfusion threshold of 70–80 g/L (plotted Hb values reflect post‐transfusion measurements). Eculizumab was initiated at 4 weeks’ gestation and increased to 1200 mg every 11 days. Due to persistent anemia and transfusion requirements, pegcetacoplan was reinitiated at 28 weeks, overlapping with eculizumab for 4 weeks (light blue), followed by dose escalation to 1080 mg three times weekly at 33 weeks to prevent peripartum breakthrough hemolysis. Pegcetacoplan therapy resulted in transfusion independence, improved hemoglobin levels, and a reduction in markers of extravascular hemolysis, with sustained stability through delivery and postpartum. Horizontal dashed lines indicate 1.0× and 1.5× upper limit of normal (ULN) for LDH. Reference intervals: lactate dehydrogenase, ≤ 214 U/L; total bilirubin, ≤ 21 µmol/L; reticulocyte count, 10–100 × 10^9^/L; hemoglobin, 115–160 g/L.

Following reinitiation of pegcetacoplan at 28 weeks’ gestation, the patient experienced rapid hematological improvement, with no transfusions required after resumption of pegcetacoplan. Hemoglobin remained stable (range 92–103 g/L) during the third trimester with concurrent declines in reticulocyte count and bilirubin, consistent with control of extravascular hemolysis (Figure 1, Table 1). Complement studies demonstrated an increase in C3 levels following pegcetacoplan reinitiation, consistent with inhibition of C3 cleavage through proximal complement blockade, and a rise in CH50 after transition from eculizumab, indicating restoration of terminal complement activity (Table 1). At 33 weeks’ gestation, despite stable hemoglobin and no evidence of intravascular breakthrough hemolysis (Figure 1), the dose of pegcetacoplan was preventatively increased to 1080 mg three times weekly to mitigate the risk of peripartum breakthrough hemolysis.

**TABLE 1:** Hematological and complement parameters by gestational and postpartum timepoints.

Timepoint	3 + 5 w	4 + 2 w^a^	7 + 3 w	10 + 5 w	14 + 5w	21 + 2 w	26 + 2 w	28 + 1 w^b^	30 + 3 w	33 w	35 + 6 w	37 w	37 + 2 w^c^	PP 1 d	PP 2 d	PP 3 d	PP 10 d	PP 6 w
Hb (g/L)	109	107	102	99	93	85	82	87	93	96	103	103	102	88	86	86	89	107
ARC (× 10^9^/L)	80	96	215	126	172	197	172	173	172	174	178	129	120	103	107.0	106.0	97	146
LDH (U/L)	153	217	220	256	213	246	243	208	205	215	208	231	210	378	351	327	280	203
Bili (µmol/L)	7	11	13	11	6	8	10	12	9	7	4	3	3	3	3	3	4	4
Hapto (g/L)	1.85	1.2	0.74	0.1	0.1	0.1				0.89	0.84	0.1					1.34	
CH50 (U/mL)		18	23	19	15	15				17	102	82	88	86.0	> 103	97		99
C3 (g/L)											4.9		4.7	4.33	4.5	4.44		
C4 (g/L)											0.43		0.37	0.34	0.35	0.36		

*Note*: Serial laboratory measurements are shown across pregnancy and postpartum (PP) days/weeks, including hemoglobin (Hb, g/L), absolute reticulocyte count (ARC, × 10⁹/L), lactate dehydrogenase (LDH, U/L), total bilirubin (Bili, µmol/L), haptoglobin (Hapto, g/L), CH50 (U/mL), C3 (g/L), and C4 (g/L). Values are presented chronologically using gestational age formatted as weeks + days (e.g., 37 + 2 w denotes 37 weeks and 2 days of gestation). Pegcetacoplan was reinitiated at 28 weeks’ gestation following inadequate response to eculizumab, with dose escalation at 33 weeks and continuation through the postpartum period. Reference intervals: hemoglobin, 115–160 g/L; reticulocyte count, 10–100 × 10^9^/L; LDH, ≤ 214 U/L; total bilirubin, ≤ 21 µmol/L; haptoglobin, 0.3–2.0 g/L; CH50, 42–95 U/mL; C3, 0.9–1.8 g/L; C4, 0.1–0.4 g/L.

^a^Date of eculizumab initiation; reinitiation.

^b^Date of pegcetacoplan reinitiation.

^c^Date of delivery by scheduled cesarean section.

The pregnancy was complicated by maternal obesity (BMI 38 kg/m^2^), gestational diabetes, and fetal growth above the 95th percentile. Given risk factors for prolonged labor, the patient elected to undergo a scheduled cesarean delivery at 37 weeks’ gestation, with dalteparin held 24 h prior to the procedure to allow for spinal anesthesia. She had an uncomplicated cesarean delivery of a healthy male infant weighing 3.74 kg with normal Apgar scores. Consecutive doses of pegcetacoplan 1080 mg were administered on postpartum Days 0–3 to prevent breakthrough hemolysis. Postpartum course was uncomplicated; the patient expressed relief at the birth of a healthy baby. She and her baby were discharged on postpartum Day 3. Pegcetacoplan 1080 mg three times weekly was continued for 6 weeks postpartum along with prophylactic anticoagulation, before returning to a twice‐weekly regimen. The patient initially breastfed during birth hospitalization but elected not to continue breastfeeding after discharge. The infant has demonstrated normal growth and development, with no complications observed at 10‐month follow‐up.

### 3.2 Pharmacokinetic Analysis

Maternal plasma concentrations of pegcetacoplan remained within expected therapeutic range, consistently near or exceeding the effective concentration for maximum hemoglobin response (EC_99_ = 597 µg/mL) on the day of delivery (Table 2) and across postpartum Days 1–3. Pegcetacoplan was undetectable in all three cord blood samples (< 10 µg/mL), with no identifiable mass spectrometry peaks, indicating no measurable transplacental drug transfer. Pegcetacoplan was likewise not detected in breast milk, with concentrations below the lower limit of quantification (< 20 µg/mL, Table 2).

**TABLE 2:** Pegcetacoplan concentrations in maternal plasma, cord blood, and breast milk.

	Concentration (µg/mL)		
	Sample 1	Sample 2	Sample 3
Maternal plasma	719	625	587
Cord blood plasma	< LLOQ	< LLOQ	< LLOQ
Breast milk	< LLOQ	< LLOQ	

*Note*: Pegcetacoplan concentrations (µg/mL) were measured using a validated LC‐MS/MS assay (lower limit of quantification [LLOQ]: 10 µg/mL for plasma; 20 µg/mL for breast milk). Maternal plasma Sample 1 was collected in the morning prior to scheduled cesarean delivery; Samples 2 and 3 were obtained later the same day following delivery. Cord blood Samples 1–3 were independently collected from the umbilical vein at delivery. Breast milk Samples 1 and 2 were collected on postpartum Days 1 and 2, respectively.

## 4 Discussion

This case provides the first pharmacokinetic data on pegcetacoplan use in pregnancy, demonstrating undetectable fetal/neonatal exposure despite sustained maternal drug levels in the therapeutic range. In a patient with hemolytic PNH and inadequate hematological response to eculizumab, conception on pegcetacoplan was not associated with adverse pregnancy outcomes, and third‐trimester reinitiation resulted in rapid hematological improvement, transfusion independence, and an uncomplicated peripartum course. Management required close collaboration between obstetrics and hematology given the high‐risk nature of pregnancy in PNH.

Pegcetacoplan has shown superior efficacy over eculizumab in patients with PNH with persistent anemia despite C5 inhibition [9], yet human pregnancy data are limited to a single case report [13], which did not include pharmacokinetic analysis. Preclinical studies in cynomolgus monkeys have shown minimal placental and breast milk transfer at therapeutic exposures, with no evidence of maternal toxicity or teratogenicity, though increased fetal loss was observed at higher exposures (2.9 × human AUC) [14]. In our case, pegcetacoplan was undetectable in all cord blood and breast milk samples, despite maternal plasma levels near or exceeding the EC_99_ for hemoglobin response. These findings suggest minimal to no placental transfer in the late third trimester and no detectable lactational transfer at therapeutic doses. These observations are consistent with the physicochemical properties of pegcetacoplan, a large (43.5 kDa), pegylated, and highly hydrophilic cyclic peptide (log *P* = −8.0), expected to substantially limit its ability to cross the placenta or mammary epithelium via passive diffusion. Given its molecular size, polarity, and absence of known active transport mechanisms, pegcetacoplan is unlikely to enter the fetal circulation or breast milk at clinically meaningful levels.

In addition to novel pharmacokinetic data, this report offers an informative contrast to the only other published case of pegcetacoplan use during pregnancy. In that case, pegcetacoplan was administered throughout gestation, which was complicated by abruptio placentae and breakthrough hemolysis at 30 weeks, necessitating an emergency cesarean delivery and eculizumab rescue [13]. By contrast, in our case, pegcetacoplan was reintroduced selectively in the third trimester, with pre‐emptive escalation to three times weekly dosing [15] at 33 weeks’ gestation to mitigate the risk of peripartum breakthrough hemolysis. This strategy was associated with sustained hematologic control, absence of breakthrough hemolysis, and an uncomplicated scheduled full‐term cesarean delivery without maternal or neonatal complications. The addition of pharmacokinetic analysis provides the first direct evidence of absent fetal and neonatal exposure to pegcetacoplan at therapeutic doses, offering important data to help inform future risk‐benefit decisions.

This report has limitations. Findings are limited to a single patient, to partial first‐ and full third‐trimester exposure, and to a limited number of pharmacokinetic timepoints. Pediatric follow‐up beyond 10 months is not yet available. Accordingly, extrapolation to the use of pegcetacoplan throughout earlier gestation and in other clinical contexts should be made with caution.

This report provides the first clinical and pharmacokinetic evidence suggesting that third‐trimester use of pegcetacoplan may be a viable treatment option in select pregnant patients with PNH with suboptimal response to C5 inhibitors. Like eculizumab, whose efficacy and safety in pregnancy were established through accumulating registry data [6], confirmation in larger cohorts will be essential to support the use of pegcetacoplan and address the unmet need for expanded treatment options for PNH in pregnancy.

## Author Contributions

B.C.Y. designed the study, cared for the patient, collected and analyzed data, and wrote the first draft of the manuscript. C.J.P. and I.C.Y. designed the study, cared for the patient, collected and analyzed data, and critically reviewed the manuscript. B.D.H., R.S., C.C.H., and M.N. collected and analyzed data and critically reviewed the manuscript. B.d.V., F.G.B., and S.P. cared for the patient, collected data, and critically reviewed the manuscript.

## Funding

This study was supported by an Investigator‐Sponsored Study (ISS) grant from Swedish Orphan Biovitrum (Sobi). Sobi had no involvement in the study design, data collection, analysis, interpretation, or reporting.

## Consent

Written informed consent was obtained from the patient for participation and for publication of this reports.

## Conflicts of Interest

B.C.Y. reports consulting and speaker fees from Alexion, Novartis, and Sobi. C.J.P. reports consulting fees from Alexion, Amgen, BioCryst, Novartis, Regeneron, Roche, and Sobi, and speaker fees from Alexion, Amgen, Novartis, and Sobi. I.C.Y. reports consulting and speaker fees from Novartis and Sobi. R.S. reports consulting fees from Novartis.

## Data Availability

The data that support the findings of this study are available on request from the corresponding author. The data are not publicly available due to privacy or ethical restrictions.
